# Efficacy and safety of intravenous ferric carboxymaltose in the treatment of Restless Legs Syndrome: a systematic review and meta-analysis

**DOI:** 10.3389/fneur.2024.1503342

**Published:** 2025-01-07

**Authors:** Ayesha Khan, Harsh Kumar, Kuldeep Dalpat Rai, Anzel Saeed, Jawad Ishtiaq, Muhammad Tanveer Alam, Sakshi Chawla, Md Ariful Haque

**Affiliations:** ^1^Department of Medicine, Dow University of Health Sciences, Karachi, Pakistan; ^2^Department of Public Health, Atish Dipankar University of Science and Technology, Dhaka, Bangladesh; ^3^Voice of Doctors Research School, Dhaka, Bangladesh

**Keywords:** Restless Legs Syndrome, Willis-Ekbom Disease, ferric carboxymaltose, iron therapy, neuromuscular disorder

## Abstract

**Introduction:**

Restless Legs Syndrome (RLS), also known as Willis-Ekbom Disease (WED), is a sensorimotor disorder characterized by an uncontrollable urge to move the legs, typically accompanied by discomfort. Low iron levels, pregnancy, and age are some identified risk factors. RLS is treated using various pharmacological options, including dopamine agonists, benzodiazepines, anticonvulsants, opioids, and bupropion. Iron supplementation, particularly with intravenous Ferric carboxymaltose (FCM), has gained attention due to the role of iron deficiency in RLS pathophysiology. This meta-analysis evaluates the efficacy and safety of FCM in treating RLS symptoms.

**Materials and methods:**

A systematic review and meta-analysis were conducted following the PRISMA guidelines, using databases such as PubMed, Google Scholar, and Cochrane. Studies involving intravenous FCM in patients diagnosed with RLS were included. Statistical analysis was performed using Review Manager 5.4.

**Results:**

Seven studies involving 539 participants were analyzed. FCM significantly reduced IRLS scores (WMD = −5.77; 95% CI = [−8.85, −2.70]; *p* = 0.0002) and improved VAS and SF-36 scores compared to placebo. However, FCM did not significantly improve RLS quality of life scores. Adverse events were more common in the FCM group, particularly nausea, but no significant differences were found for severe adverse events.

**Conclusion:**

In conclusion, intravenous ferric carboxymaltose significantly reduces Restless Legs Syndrome symptoms, especially in patients with confirmed iron deficiency. The treatment appears generally well-tolerated, with adverse effects being manageable. However, further long-term studies are needed to fully assess the safety profile and confirm sustained symptom improvement in a broader population.

**Systematic review registration:**

https://www.crd.york.ac.uk/prospero/, identifier: CRD42024585233.

## 1 Introduction

Restless Legs Syndrome (RLS) is a neuromuscular disorder characterized by an uncomfortable and sometimes painful sensation during inactivity of the legs (1). These sensations are temporarily relieved by movement. The discomfort can manifest as crawling, tingling, or aching feelings in the legs and may cause significant disruption to sleep and daily activities. Over the last 10 years, there has been a high focus on studying Restless Legs Syndrome. Yet, there is still limited knowledge about this condition and countless individuals experience symptoms of RLS without receiving a diagnosis from healthcare professionals. A recent study found that despite 45% of patients showing moderate to severe RLS symptoms, none were diagnosed or treated (2). Only 6.2% of people experiencing RLS symptoms and seeking medical assistance stated that they had been diagnosed with RLS (3). The inadequate diagnosis given to patients with RLS highlights the lack of knowledge about this disorder.

The International RLS Study Group (IRLSSG) established the RLS standard diagnostic criteria in 2003. The key factors of RLS comprise: (a) a desire to move the legs, frequently coupled with discomfort in the legs or other areas of the body; (b) symptoms worsened during rest; (c) symptoms improved by movement and (d) symptoms that peak in the evening or night (3). There is also a 1995 IRLSSG diagnostic criteria for diagnosing RLS (4), used by studies before 2005. This criteria also suggests four essential characteristics for diagnosing the condition: (a) urge to move limbs, usually accompanied by abnormal sensations; (b) physical restlessness; (c) symptoms aggravated during rest but with temporary relief through activity and movement; and (d) symptom exacerbation in the evening or night.

Studies on the epidemiology of RLS suggest that the prevalence rates in the general population range from 1 to 15% in various populations (5–7). Previous studies have suggested that the actual prevalence of RLS could be underestimated due to patients being scared of social rejection in some cultures, not experiencing symptoms during testing, and the belief that it is a minor issue not requiring medical intervention (8). Numerous global studies show a higher occurrence of RLS in women, indicating that women are at a higher risk than men worldwide, regardless of location. A study proposed that a potential genetic pattern might contribute to women's higher prevalence of RLS (9).

The risk factors that might contribute to RLS include (a) pregnancy: studies found a higher rate of RLS in pregnant women and hypothesized that hormones might be the cause (10). (b) Low iron levels: the “Iron Hypothesis” explains the connection of low iron levels with RLS. RLS can hinder the typical age-related rise in brain iron, leading to decreased iron levels in specific brain regions and impacting brain function (11). (c) Lower socioeconomic status (12). (d) Poor health (13). (e) Old age: supported by the results from the REST study (2004), which concluded that the prevalence of RLS increased from a 1% prevalence in the 20–29 age group to a 4% prevalence in the 70–89-year-old groups. (f) Parkinson's disease, (g) psychiatric disorders, and (h) end-stage renal disease (14).

The possible treatments of RLS include dopamine agonists, benzodiazepines, anticonvulsants (such as carbamazepine or gabapentin), opioids (such as codeine or oxycodone), bupropion and iron supplementation (14). Multiple articles have covered various aspects of each of these treatments. Still, this article provides explicitly an analysis of clinical trials done to assess the efficacy and safety of the treatment of RLS with ferric carboxymaltose (FCM), an intravenous iron supplement.

## 2 Materials and methods

This systematic review and meta-analysis fully comply with the preferred reporting items for the systematic review and meta-analysis (PRISMA) 2020 statement (15). The protocol is registered in PROSPERO with protocol ID: CRD42024585233.

### 2.1 Database and literature search strategy

We performed a literature search using the following electronic databases: PubMed (MEDLINE), Google Scholar, and Cochrane Central Register of Controlled Trials (The Cochrane Library) from inception to Aug 1 2024 for all relevant articles that used “Restless Legs Syndrome,” “RLS,” “Willis -Ekbom disease,” “ferric carboxymaltose,” “ferric compounds,” “injectafer,” and “FCM.” as keywords in the title, subject heading, and text word. The detailed database search strategy is shown in Supplementary Table 1. The search results were not filtered or restricted in any way. All potentially relevant studies, articles (including undocumented data and meta-analyses), and international guidelines were searched manually, and their references were cross-searched to identify additional suitable studies. Two independent investigators (JJ and KK) evaluated the published articles, and disagreements were fully discussed to reach a consensus.

### 2.2 Selection procedure and eligibility criteria

The eligibility criteria included (1) Participants must have a diagnosis of RLS according to the international Restless Leg Syndrome Study Group (IRLS) criteria, with a score of 15 or higher. (2) The studies must evaluate clinical outcomes related to RLS, such as changes in IRLS scores, VAS pain levels, or quality of life (QoL) metrics. (3) The intervention arm must involve IV FCM as the iron replacement therapy for managing RLS symptoms.

Exclusion criteria consisted of (1) non-randomized studies, including case reports and observational research due to concerns about bias, (2) no control group in the trial, and (3) participants with RLS, which is secondary to any other disease condition. Supplementary Table 2 provides information on the study characteristics of the included studies.

### 2.3 Data extraction

Studies were independently screened and assessed by two reviewers (KD and JI). For each RCTS, data on study characteristics (e.g., author, year, sample size, and follow-up), patient demographics (age, sex), and intervention details (dose of IV FCM) were extracted. Information on clinical outcomes, including IRLS scores, VAS pain ratings, ferritin, Hb levels, and adverse events, was also collected. Additionally, the overall conclusion of each study regarding the effect of IV FCM on RLS was noted. Two reviewers independently extracted these data using predefined criteria. Primary authors of the selected publications were contacted when the relevant information was not reported. If the above data could not be found, items were designated as “N/A (not available).” In cases where a consensus could not be reached, the opinion of a third reviewer (AK) was sought.

### 2.4 Risk of bias assessment

**Figure 1:**
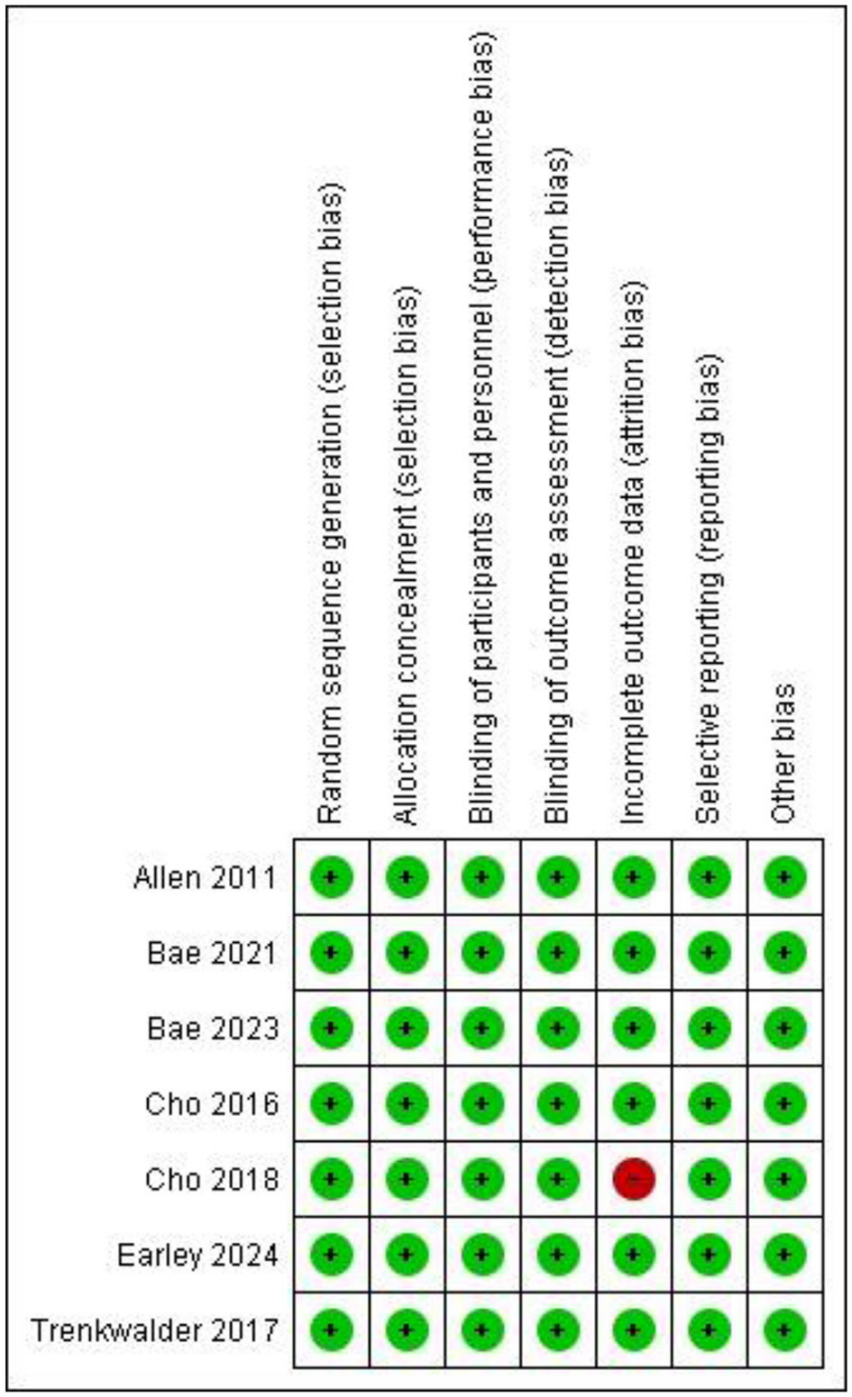
Risk of bias assessment.

The quality of included RCTs was independently assessed by two authors (AK AND AS) using RevMan 5.4.1 software according to the Cochrane Handbook, and the ROB-2 tool was used to assess the risk of bias in RCTs (10). In cases where a consensus could not be reached, the opinion of a third reviewer (SC) was sought. The risk-of-bias assessment included five items, as follows: (1) adequate sequence generation; (2) allocation concealment; (3) incomplete outcome data; (4) free of selective reporting; (5) free of other biases. The judgments were categorized as “yes” (low risk of bias), “no” (high risk of bias), or “unclear” (unclear risk of bias; Supplementary Figure 2). All analyses were based on previously published studies; thus, no ethical approval and patient consent were required. Figure 1 presents an assessment of the risk of bias.

### 2.5 Statistical analyses

The analysis was conducted using (RevMan) Review Manager Version 5 with a random effects model applied to account for variations between studies. The standard mean difference (SMD) and 95% confidence interval CI were calculated for continuous data. Relative risks (RR) or risk difference (RD) were used for dichotomous data. A *p*-value below 0.05 was considered statistically significant. Heterogeneity within the clinical trials was evaluated using the Higgins (*I*^2^) statistic, with interpretations as follows: 25–50% indicating low heterogeneity, 50–75% indicating moderate heterogeneity, and >75% indicating high heterogeneity. A sensitivity analysis was conducted to identify its source in cases of high heterogeneity.

## 3 Results

### 3.1 Article selection

**Figure 2:**
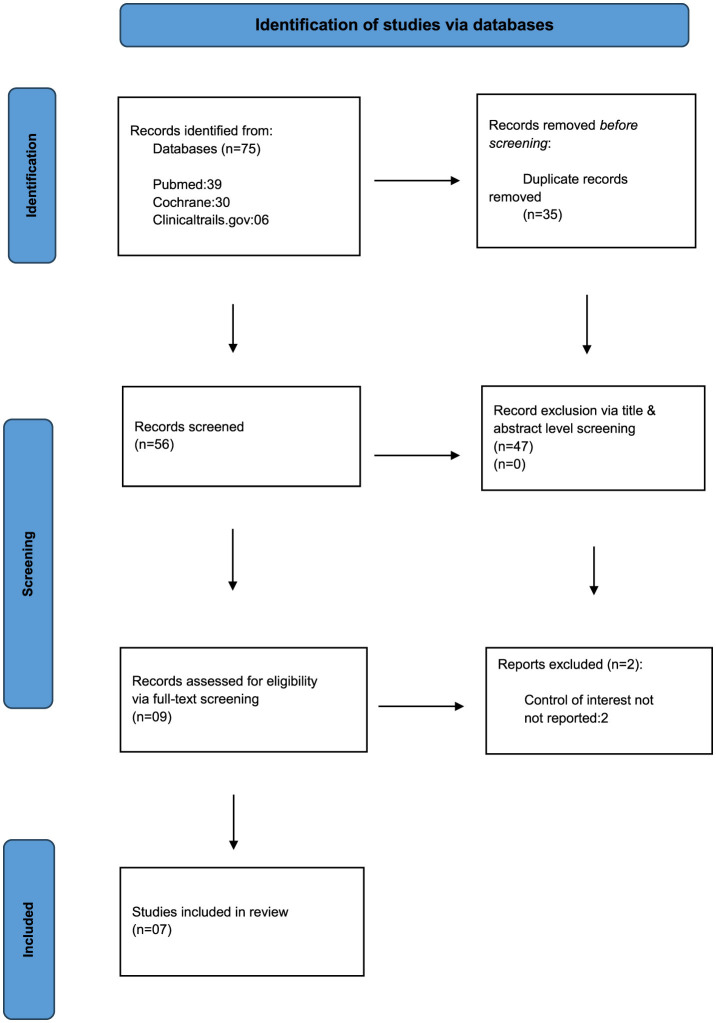
Prisma flow chart.

Our initial search led to the identification of 1,280 records, which were reduced to 967 after the removal of duplicates. After a preliminary assessment of these records, 41 articles were shortlisted for further scrutiny. Six articles were selected based on matching inclusion and exclusion criteria (17–21). Of the 41 articles scrutinized, 16 were excluded due to irrelevant outcomes, eight due to add-on therapeutic agents, nine due to different control agents, and finally, two were secondary to incomplete data being present. The selection process is summarized in Figure 2.

### 3.2 Results of the systematic review and meta-analysis

The findings of our systematic review and meta-analysis are presented below, with primary outcomes comprising IRLS change from baseline and secondary outcomes being the RLS Qol, VAS score, SF-36, and safety outcomes.

### 3.3 Primary outcome

#### 3.3.1 IRLS change from baseline

**Figure 3:**
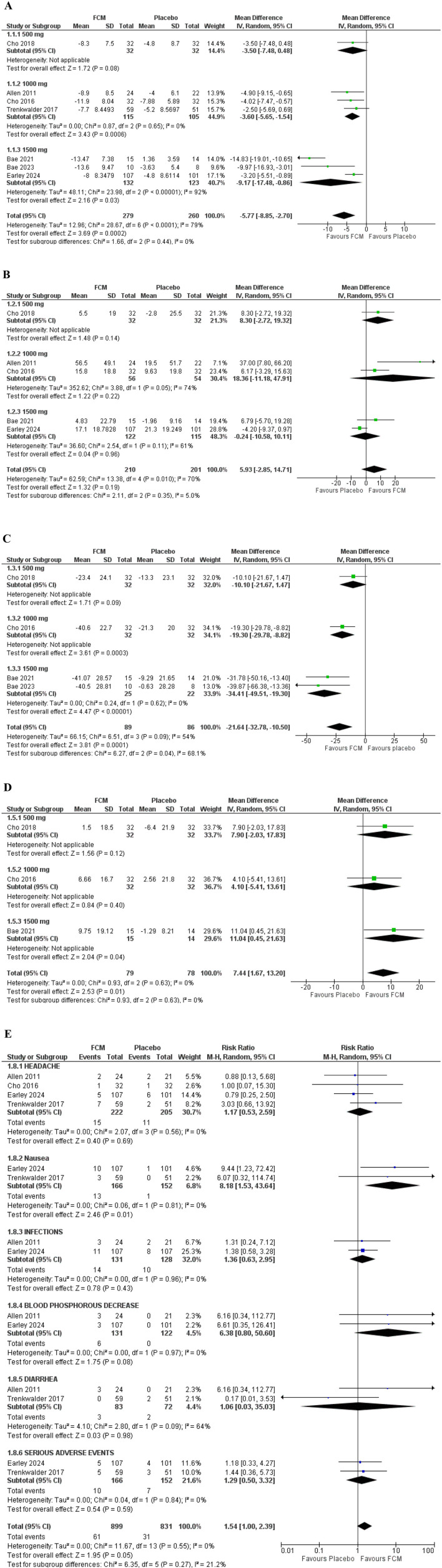
Forest plots; primary outcome **(A)** IRLS change from baseline. Secondary outcomes **(B)** RLS QOL **(C)** VAS score **(D)** SF-36 **(E)** adverse effects.

Seven studies assessed the effect of FCM on the IRLS score. The pooled analysis of data from these studies revealed that FCM elicited a significant decrease in IRLS score compared to the placebo group (WMD = −5.77; 95% CI= [−8.85, −2.70]; *p* = 0.0002). Seventy-nine percent heterogeneity was observed (Figure 3A).

### 3.4 Secondary outcomes

#### 3.4.1 RLS QOL

Five studies evaluated the RLS Quality of Life score. The pooled analysis of data from these three studies revealed that FCM elicited an insignificant improvement in QOL-PFD scores compared to the placebo group (WMD = 5.93; 95% CI = [−2.85, 14.71]; *p* = 0.19). Seventy-percent heterogeneity was observed (Figure 3B).

#### 3.4.2 VAS score

Four studies assessed the effect of FCM on VAS scores in total. The pooled data analysis from these studies revealed that FCM elicited a significant decrease in VAS score compared to the placebo group (WMD = −21.64; 95% CI = [−32.78, −10.50]; *p* = 0.0001). However, 54% heterogeneity was observed (Figure 3C).

#### 3.4.3 SF-36

Three studies evaluated the SF-36 score. The pooled analysis of data from these three studies revealed that FCM caused a significant improvement in SF-36 score compared to the placebo group (WMD = 7.44; 95% CI = [1.67, 13.20]; *p* = 0.01). There was 0% heterogeneity recorded (Figure 3D).

### 3.5 Safety outcomes

#### 3.5.1 Adverse events

##### 3.5.1.1 Headache

Four studies assessed the risk ratio of headaches in individuals with RLS treated with FCM compared to those treated with placebo. The risk ratio for headache was 1.17 (95% CI = [0.53, 2.59]; *p* = 0.69; *I*^2^ = 0%), indicating a non-significant difference between the two groups (Figure 3E).

##### 3.5.1.2 Nausea

Two studies assessed the risk ratio of nausea in individuals with RLS treated with FCM compared to those treated with placebo. The risk ratio for nausea was 8.18 (95% CI = [1.53, 43.64]; *p* = 0.01; *I*^2^ = 0%), indicating a significantly higher incidence of nausea in the FCM group compared to placebo.

##### 3.5.1.3 Infections

Two studies assessed the risk ratio of infections in individuals with RLS treated with FCM compared to those treated with placebo. The infection risk ratio was 1.36 (95% CI = [0.63, 2.95]; *p* = 0.43; *I*^2^ = 0%), indicating a non-significant difference between the two groups.

##### 3.5.1.4 Blood phosphorous decrease

Two studies assessed the risk ratio of Blood phosphorus decrease in individuals with RLS treated with FCM compared to those treated with placebo. The risk ratio for Blood phosphorus decrease was 6.38 (95% CI = [0.80, 50.60]; *p* = 0.08; *I*^2^ = 0%), indicating a non-significant difference between the two groups.

##### 3.5.1.5 Diarrhea

Two studies assessed the risk ratio of Diarrhea in individuals with RLS treated with FCM compared to those treated with placebo. The risk ratio for Diarrhea was 1.06 (95% CI = [0.03, 35.03]; *p* = 0.98; *I*^2^ = 0%), indicating a non-significant difference between the two groups.

##### 3.5.1.6 Serious adverse effects

Two studies assessed the risk ratio of serious adverse effects in individuals with RLS treated with FCM compared to those treated with placebo. The risk ratio for Serious adverse effects was 1.29 (95% CI = [0.50, 3.32]; *p* = 0.59; *I*^2^ = 0%), indicating a non-significant difference between the two groups.

### 3.6 Sensitivity analysis

#### 3.6.1 IRLS score

A sensitivity analysis evaluated heterogeneity by excluding the Bae 21 study. This resulted in a reduction of heterogeneity to 0% (WMD = −3.70; 95% CI = [−5.10, −2.30]; *p* < 0.00001; *I*^2^ = 0%), illustrated by Supplementary Figure 2.

#### 3.6.2 RLS QOL

We conducted a sensitivity analysis to assess heterogeneity by excluding the Early 21 study. This exclusion reduced the heterogeneity to 24%, and a significant mean difference was observed between the two groups in the RLS-QoL score (WMD = 8.81; 95% CI = [1.49, 16.12]; *p* = 0.02; *I*^2^ = 24%), illustrated by Supplementary Figure 3.

## 4 Discussion

This systematic review and meta-analysis includes most studies and patients of any previous study comparing FCM to placebo in patients with RLS. This meta-analysis includes seven studies with 539 participants, of which 279 patients received FCM, and 260 received a placebo. The pooled data analysis showed that compared to a placebo, FCM is a more effective intervention for alleviating the symptoms of Restless Legs Syndrome.

RLS has been associated with dopaminergic dysfunction in the brain. However, contrary to common belief, it is not solely attributable to this; the underlying pathophysiology is more intricate. Iron deficiency has been implicated in RLS, and it is primarily associated with brain iron deficiency rather than systemic iron deficiency, which has been proved by low iron levels in neuropathological samples, brain imaging studies, and cerebrospinal fluid analysis (16). Intravenous iron supplementation has emerged as an effective treatment for iron deficiency without causing harmful iron buildup in the brain. Studies in iron-deficient mice showed that this method could increase iron levels in the substantia nigra, a brain region important for movement, without affecting iron levels in other parts of the brain (17).

The pooled data analysis showed that treating individuals with FCM is associated with a significant reduction in IRLS scores compared to a placebo. These results were consistent with previous studies by Avni et al.; however, their findings were based on limited studies and a small patient population (18). With new literature available with a greater population size, our analysis showed that FCM decreased the IRLS score by 5 points (*p* < 0.05). A subgroup analysis using different dosages showed that 1,500 mg was associated with a more significant improvement in the IRLS scores than 500 and 1,000 mg.

Our analysis also showed that FCM was associated with significantly improved VAS and SF-36 scores. VAS is a commonly used tool to assess pain in chronic conditions (19), and SF-36 is a multi-item scale that evaluates several health concepts and general health perceptions, such as limitations in physical and social activities (20). This is the first meta-analysis that has compared these outcomes in individuals taking FCM for RLS.

On the other hand, the analysis of the RLS-QOL score outcome did not show any significant difference between the FCM and placebo groups. These findings were inconsistent with a previous meta-analysis by Avni et al. which showed that compared to placebo, FCM significantly increased the Qol score by 8 points (18). More studies are warranted to establish a more conclusive result.

To understand the safety profile of the drug, an analysis of adverse events such as headache, nausea, and diarrhea was done. Compared to the placebo, FCM showed no significant increase in adverse events except for nausea. These findings were similar to a retrospective study by Park et al., which showed no adverse effects with IV FCM infusion (21).

Although the systemic review and meta-analysis show promising results for FCM use in individuals with RLS, there were some limitations. The follow-up duration varied among some studies. Two of the included studies reported data after 4 weeks, while the others had a follow-up duration of 6 weeks. This change might impact our outcomes to a certain degree. Furthermore, the meta-analysis was conducted under the assumption that the baseline characteristics of all studies were similar. Some studies included a higher proportion of patients with anemia, which could have influenced the outcomes. Therefore, further studies are needed to understand the efficacy of FCM in RLS individuals.

## Data Availability

The original contributions presented in the study are included in the article/Supplementary material, further inquiries can be directed to the corresponding author.
