# Supplementary material for: Efficacy and safety of intravenous ferric carboxymaltose in the treatment of Restless Legs Syndrome: a systematic review and meta-analysis
Source: Front Neurol. 2025 Jan 7;15:1503342. doi: 10.3389/fneur.2024.1503342 (PMC11746117; doi:10.3389/fneur.2024.1503342)
Supplement: Supplementary file 1 [file Data_Sheet_1.docx]

**Index**

1. Search strategy**- Table 1**
2. Risk of bias graph- **Figure 1**
3. Baseline Characteristics- **Table 2**
4. Forest plots of sensitivity analysis-**Figure 2 and 3**
5. References of the included studies

**Supplementary table 1:** Search strategy.

| Search engine | Search strategy | results |
| --- | --- | --- |
| Pubmed | ((((((Restless Legs syndrome) OR (RLS)) OR (Willis -Ekbom disease)) AND (ferric carboxymaltose)) OR (ferric compounds)) OR (injectafer)) OR (FCM)  (("restless legs syndrome"[MeSH Terms] OR ("restless"[All Fields] AND "legs"[All Fields] AND "syndrome"[All Fields]) OR "restless legs syndrome"[All Fields] OR "RLS"[All Fields] OR ("restless legs syndrome"[MeSH Terms] OR ("restless"[All Fields] AND "legs"[All Fields] AND "syndrome"[All Fields]) OR "restless legs syndrome"[All Fields] OR ("willis"[All Fields] AND "ekbom"[All Fields] AND "disease"[All Fields]) OR "willis ekbom disease"[All Fields])) AND ("ferric carboxymaltose"[Supplementary Concept] OR "ferric carboxymaltose"[All Fields])) OR ("ferric compounds"[MeSH Terms] OR ("ferric"[All Fields] AND "compounds"[All Fields]) OR "ferric compounds"[All Fields]) OR ("ferric carboxymaltose"[Supplementary Concept] OR "ferric carboxymaltose"[All Fields] OR "injectafer"[All Fields]) OR "FCM"[All Fields] | 478 |
| Google scholar | ((((((Restless Legs syndrome) OR (RLS)) OR (Willis -Ekbom disease)) AND (ferric carboxymaltose)) OR (ferric compounds)) OR (injectafer)) OR (FCM) | 321 |
| Cochrane | ((((((Restless Legs syndrome) OR (RLS)) OR (Willis -Ekbom disease)) AND (ferric carboxymaltose)) OR (ferric compounds)) OR (injectafer)) OR (FCM) | 415 |

**Supplementary Figure 1: Risk of bias graph**


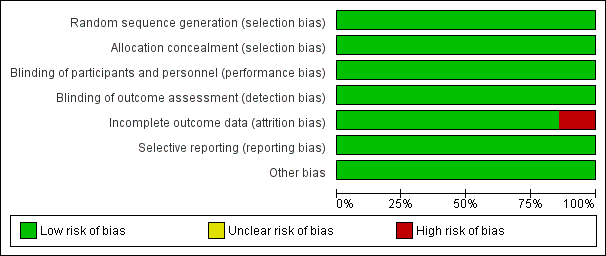


**Supplementary Table 2: Baseline Characteristics of included studies**

| STUDY | INTERVENTION | INCLUDED PATIENTS | HAEMATOLOGICAL INCLUSION CRITERIA | OTHER MEDICATIONS FOR RLS | N PATIENTS RANDOMIZEDmn6 | TIME OF EFFICACY MEASUREMENT | FOLLOW UP DURATION | AGE | % FEMALE | BASELINE IRLS SCORE | MEAN BASELINE FERRITIN | MEAN BASELINE Hb | VAS | IRLS QoL |
| --- | --- | --- | --- | --- | --- | --- | --- | --- | --- | --- | --- | --- | --- | --- |
| Allen^1^ 2011 | IVFCM500mg, 2timesover 2weeks | IRLSscore≥15 | ferritin<45% | Notallowed | 24 | 4 WEEKS | 12 WEEKS | 49.5±11.4 | 70% | 25.0±5.8 | 28.1±22.9 | Not reported |  |  |
|  | Placebo |  |  |  | 22 |  |  | 54.8±13.6 | 52% | 24.2±5.5 | 24.8±20.2 |  |  |  |
|  |  |  |  |  |  |  |  |  |  |  |  |  |  |  |
| Bae^2^ 2021 | IV FCM 1500mg, 1000 mg at day 1 and 500 mg 1 week later | IRLSscore≥15 | hemoglobin <12 g/dL, either ferritin <20 ng/ ml or ferritin <100 ng/ml with transferrin saturation <18% | Notallowed | 15 | 6 WEEKS | 52 WEEKS | 48.00 ± 13.50 | 13 (86.67%) | 25.60 ± 7.35 | 5.82 ± 5.05 | 10.27 ± 22.41 | 67.67 ± 25.56 | 67.00 ± 22.06 |
|  | Placebo |  |  |  | 14 |  |  | 47.71 ± 6.04 | 13 (92.86%) | 25.21 ± 5.58 | 7.91 ± 9.84 (13) | 10.46 ± 0.94 | 65.36 ± 26.64 | 65.00 ± 17.47 |
|  |  |  |  |  |  |  |  |  |  |  |  |  |  |  |
| Bae^3^ 2023 | IV FCM 1500mg, 1000 mg at day 1 and 500 mg 1 week later | IRLSscore≥15 | serum hemoglobin <12 g/dL and either ferritin <20 ng/ml or ferritin <100 ng/ml with transferrin saturation <18% | Notallowed | 10 | 6 WEEKS | not given | 42.10 ± 7.11 | 9 (90.0%) | 23.70 ± 8.04 | 3.73 ± 2.56 | 10.16 ± 1.55 | 62.00 ± 26.58 |  |
|  | Placebo |  |  |  | 8 |  |  | 46.25 ± 4.53 | 8 (100.0%) | 24.75 ± 7.21 | 3.05 ± 0.91 | 10.08 ± 1.01 | 55.63 ± 27.70 |  |
|  |  |  |  |  |  |  |  |  |  |  |  |  |  |  |
| Cho^4^ 2016 | IVFCM 1000mg,once | IRLSscore≥15 | ferritin12μg/dL,or TSAT <45% | Notallowed | 32 | 6 WEEKS | 30 WEEKS | 49.7±13.7 | 81% | 27.4±4.03 | 24.8±20.2 | 13.3±1.42 |  |  |
|  | Placebo |  |  |  | 32 |  |  |  | 75% | 28.0±5.16 | 53.5±41.8 | 13.5±1.11 |  |  |
|  |  |  |  |  |  |  |  |  |  |  |  |  |  |  |
| Cho^5^ 2018 | IVFCM500mg, once | IRLSscore≥15 | ferritin12μg/dL,or TSAT <45% | Notallowed | 32 | 6 WEEKS | 30 WEEKS | 47.3±13.3 | 81% | 28.1±5.7 | 50.7±40.8 | 13.5±1.2 |  |  |
|  | Placebo |  |  |  | 32 |  |  | 51.5±12.0 | 81% | 27.3±5.3 | 70.2±59.5 | 13.3±0.9 |  |  |
|  |  |  |  |  |  |  |  |  |  |  |  |  |  |  |
| Earley^6^ 2024 | IV FCM 1500mg, 750 mg at day 1 and 750 mg 5 days later | IRLSscore≥15 | excluded if ferritin level ≥300 ng/mL, transferrin saturation ≥45%, hemoglobin level that exceeded the upper limit of normal | Notallowed | 107 | 6 WEEKS | 12 MONTHS | 58.3 ± 12.3 | 71 (66.4%) | - | 64.8 ± 51.2 | Not reported |  |  |
|  | Placebo |  |  |  | 101 |  |  | 56.9 ± 13.7 | 69 (68.3%) |  | 66.5 ± 56.6 |  |  |  |
|  |  |  |  |  |  |  |  |  |  |  |  |  |  |  |
| Trenkwalder^7^ 2017 | IVFCM 1000mg,once | IRLSscore≥15 | Ferritin<75orferritin 75–300andTSAT<20%, Hb<11.5/12.5(male/ female) | Not specified | 59 | 4 WEEKS | 12 WEEKS | 53.0±15.7 | 81% | 25.9±5.65 | 41.93±34.55 | Not reported |  |  |
|  | Placebo |  |  |  | 51 |  |  | 55.5±15.9 | 82% | 26.0±5.78 | 48.85±45.95 |  |  |  |

**Supplementary Figure 2: Forest plot, sensitivity analysis of IRLS Score**


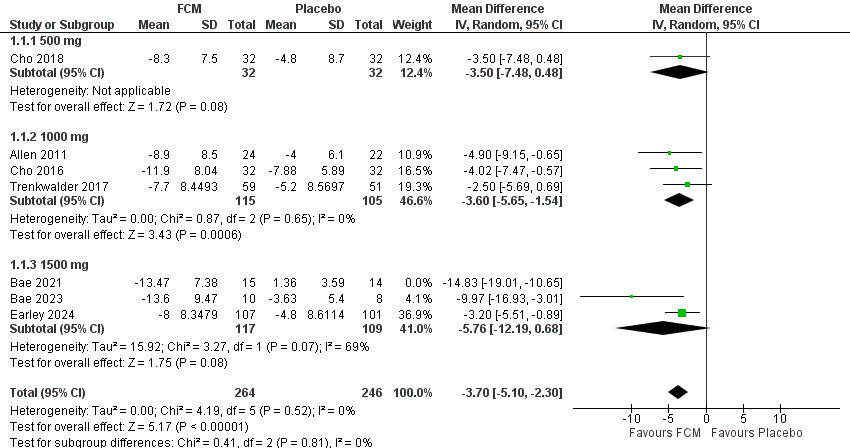


**Supplementary Figure 3: Forest plot, sensitivity analysis of RLS QOL**


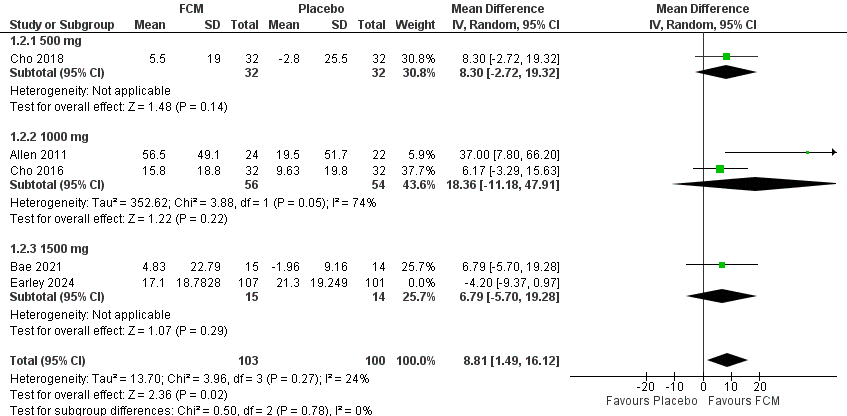


**References :**

1. Allen, R. P., Adler, C. H., Du, W., Butcher, A., Bregman, D. B., & Earley, C. J. (2011). Clinical efficacy and safety of IV ferric carboxymaltose (FCM) treatment of RLS: a multi-centred, placebo-controlled preliminary clinical trial. *Sleep medicine*, *12*(9), 906–913. <https://doi.org/10.1016/j.sleep.2011.06.009>
2. Bae, H., Cho, Y. W., Kim, K. T., Allen, R. P., & Earley, C. J. (2021). Randomized, placebo-controlled trial of ferric carboxymaltose in restless legs syndrome patients with iron deficiency anemia. *Sleep medicine*, *84*, 179–186. <https://doi.org/10.1016/j.sleep.2021.05.036>
3. Bae, H., Cho, Y. W., Kim, K. T., Li, X., & Earley, C. J. (2023). Ferric carboxymaltose effects on restless legs syndrome and on brain iron in patients with iron deficiency anemia. *Sleep medicine*, *109*, 128–131. <https://doi.org/10.1016/j.sleep.2023.06.023>
4. Cho, Y. W., Allen, R. P., & Earley, C. J. (2016). Clinical efficacy of ferric carboxymaltose treatment in patients with restless legs syndrome. *Sleep medicine*, *25*, 16–23. <https://doi.org/10.1016/j.sleep.2016.06.021>
5. Cho, Y. W., Allen, R. P., & Earley, C. J. (2018). Efficacy of ferric carboxymaltose (FCM) 500 mg dose for the treatment of Restless Legs Syndrome. *Sleep medicine*, *42*, 7–12. <https://doi.org/10.1016/j.sleep.2017.11.1134>
6. Earley, C. J., García-Borreguero, D., Falone, M., & Winkelman, J. W. (2024). Clinical efficacy and safety of intravenous ferric carboxymaltose for treatment of restless legs syndrome: a multicenter, randomized, placebo-controlled clinical trial. *Sleep*, *47*(7), zsae095. <https://doi.org/10.1093/sleep/zsae095>
7. Trenkwalder, C., Winkelmann, J., Oertel, W., Virgin, G., Roubert, B., Mezzacasa, A., & FCM-RLS Study Investigators (2017). Ferric carboxymaltose in patients with restless legs syndrome and nonanemic iron deficiency: A randomized trial. *Movement disorders : official journal of the Movement Disorder Society*, *32*(10), 1478–1482. <https://doi.org/10.1002/mds.27040>
